# Novel Three-Dimensional and Non-Invasive Diagnostic Approach for Distinction between Odontogenic Keratocysts and Ameloblastomas

**DOI:** 10.3390/dj11080193

**Published:** 2023-08-11

**Authors:** Juergen Taxis, Natascha Platz Batista da Silva, Elisabeth Grau, Gerrit Spanier, Felix Nieberle, Michael Maurer, Steffen Spoerl, Johannes K. Meier, Tobias Ettl, Torsten E. Reichert, Nils Ludwig

**Affiliations:** 1 Department of Cranio- and Maxillofacial Surgery, University Hospital Regensburg, Franz-Josef-Strauß-Allee 11, 93053 Regensburg, Germany; gerrit.spanier@ukr.de (G.S.); felix.nieberle@stud.uni-regensburg.de (F.N.); michael.maurer@ukr.de (M.M.); steffen.spoerl@ukr.de (S.S.); johannes.meier@ukr.de (J.K.M.); tobias.ettl@ukr.de (T.E.); torsten.reichert@ukr.de (T.E.R.); nils.ludwig@ukr.de (N.L.); 2 Department of Radiology, University Hospital Regensburg, Franz-Josef-Strauß-Allee 11, 93053 Regensburg, Germany; natascha.platz-batista-da-silva@ukr.de; 3 Department of Oral and Maxillofacial Surgery, Leipzig University Medical Center, Liebigstraße 12, 04103 Leipzig, Germany; elisabeth.grau@medizin.uni-leipzig.de

**Keywords:** odontogenic keratocyst, ameloblastoma, non-invasive differential diagnosis, CT, CBCT, hounsfield units differentiation, gray scale values differentiation

## Abstract

Aim of this study was to demonstrate the diagnostic ability to differentiate odontogenic keratocysts (OKCs) from ameloblastomas (AMs) based on computed tomography (CT) or cone beam computed tomography (CBCT) scans. Preoperative CT and CBCT scans from 2004 to 2019 of OKCs and AMs were analyzed in 51 participants. Lesions were three-dimensionally (3D) assessed and Hounsfield units (HU) as well as gray scale values (GSV) were quantified. Calculated HU spectra were compared within the same imaging modalities using unpaired *t*-tests and correlated with participants characteristics by calculating Pearsons correlation coefficients. Within the CT scans, AMs had highly significantly higher HU values compared to OKCs (43.52 HU and 19.79 HU, respectively; *p* < 0.0001). Analogous, within the CBCT scans, AMs had significantly higher GSV compared to OKCs (−413.76 HU and −564.76 HU, respectively; *p* = 0.0376). These findings were independent from participants’ gender and age, anatomical site, and lesion size, indicating that the HU- and GSV-based difference reflects an individual configuration of the lesion. HU and GSV spectra calculated from CT and CBCT scans can be used to discriminate between OKCs and AMs. This diagnostic approach represents a faster and non-invasive option for preoperative diagnosis of such entities and has potential to facilitate therapeutic decision making.

## 1. Introduction

Odontogenic keratocysts (OKCs) and ameloblastomas (AMs) are two types of benign lesions that can develop in any part of the jaw [1,2]. OKCs are epithelial-lined cysts that account for up to 20% of cystic jaw lesions and are often associated with a nevoid basal cell carcinoma syndrome [1,2,3,4]. In contrast, AMs are usually slow-growing epithelial odontogenic tumors that are among the most common jaw tumors [1,2,5]. Overall, both lesions can occur at any age and are more common in the posterior part of the mandible [1,5,6,7,8,9]. Also, both OKCs and AMs can recur after treatment, so long-term follow-up is necessary to intervene if there are signs of recurrence [10,11]. Hereby, early detection and prompt treatment are critical to achieve the best outcomes for patients with these entities.

Since OKCs and AMs are usually asymptomatically in early stages, they are often discovered as incidental findings during routine radiographic examinations [1,12]. Nevertheless, despite their benign character, OKCs and AMs can reach an enormous extent associated with destructive property and severe pain [13]. Diagnosis usually involves a combination of clinical examination, radiographs, and subsequent biopsy. Clinical examination may reveal swelling or other abnormalities in the jaw or surrounding tissues. Radiographs, such as panoramic radiograph, cone beam computed tomography (CBCT), or a computed tomography (CT) scan can provide more accurate information about the size and location of the lesion. However, radiology-based primary discrimination between OKCs and AMs is challenging due to their similar appearance on imaging [14]. Therefore, a biopsy is required in most cases to confirm the diagnosis and to guide further therapy [7]. In the case that an extensive OKC is diagnosed histopathologically, the initial treatment is considered to be a marsupialisation in order to reduce the size of the lesion over a period of time, spare closely adjacent structures, and perform enucleation on a small-size lesion [1]. However, after histopathologic diagnosis of AMs the immediate resection of the lesion is required to counteract progression or recurrence [1,15].

Since, on the one hand, a biopsy is associated with a certain loss of time and does not necessarily yield representative tissue and, on the other hand, a reliable radiological confirmation of the diagnosis is sometimes not possible on the basis of, for example, the rapidly available panoramic radiograph, the search for a non-invasive imaging option appears to be reasonable. In previous work, attempts have been made to differentiate between both lesions using CT and MRI scans [16,17,18,19,20]. Further studies investigated the possibility of using machine learning to distinguish between these two entities within panoramic radiographs or even CT scans [12,21]. Attempts have also been made to differentiate such lesions on the basis of morphology or radiographic features [7,22,23].

For different tissue types and fluids, specific ranges of Hounsfield units (HU) can be given as an expression of the attenuation coefficient within a CT [24,25]. Thus, HU might be a potential parameter to discriminate between OKCs and AMs, since both entities might include a specific spectrum of HU due to their individual configuration in three-dimensional (3D) imaging modalities. To the best of our knowledge, only one publication exists that addresses the differentiation of OKCs and AMs based on HU within CT scans [26]. In contrast, no studies were found with regards to CBCTs. Therefore, the aim of this study was to investigate whether HU or gray scale values (GSV) can be used to discriminate OKCs from AMs in CT as well as CBCT scans, demonstrating a novel noninvasive diagnostic approach. Thus, the respective null hypothesis is that the differentiation of OKCs from AMs based on HUs in CT and GSV in CBCT is not possible.

## 2. Materials and Methods

### 2.1. Participant Selection and Data Collection

**Figure 1:**
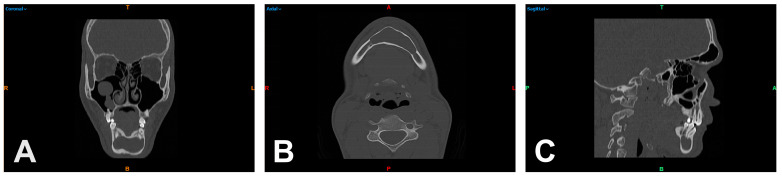
Multiplanar reformation of the midface including both jaws (bone windows) of a participant with an odontogenic keratocyst in the mandibular front in coronal (**A**), axial (**B**), and sagittal reformation (**C**). R = right; T = top; L = left; colored B = bottom; colored A = anterior; P = posterior.

In this study, a participant population was evaluated between the years 2004 and 2019, in which surgical treatment was performed due to an OKC or an AM in the maxillary and mandibular region. The initial cohort, from which histologic confirmation of the findings was available, included a total of 124 participants. Clinical data such as age, sex, histologic findings, location of the entity, and used imaging modality were included in the analysis. The most important criterion was the presence of a preoperative high-resolution CT scan or, alternatively, a CBCT scan. The default settings were used to create the CT and CBCT scans. CT scans were performed using a SOMATOM Definition Flash (Siemens Healthcare GmbH, Erlangen, Germany) with 120 kV and 112–162 mAs. The scans required for DICOM data extraction and secondary measurements were coronal, axial, and sagittal reformations of the midface including both jaws (bone windows) with a slice thickness of 0.75 mm (Figure 1A–C). The CBCT scans, on the other hand, were obtained using an orangedental Master 3DS (orangedental GmbH & Co. KG, Biberach, Germany) with 90 kV, 5 mA, a slice thickness of 0.3 mm, and an FOV of 16 × 10 cm. These criteria reduced the final collective to 51 participants eligible for further segmentation and measurement of the respective HU and GSV spectrum.

### 2.2. Hounsfield Unit-Based Quantification of Lesions

**Figure 2:**
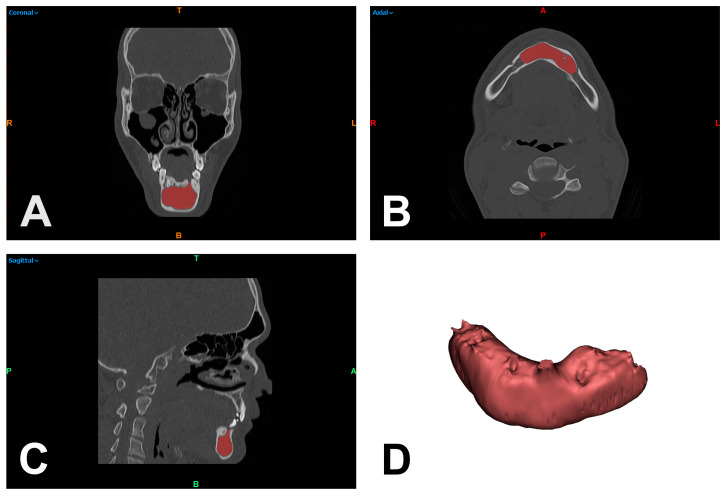
Separation of cystic structures (red area) by thresholding of Hounsfield units and virtual bone dissection in coronal (**A**), axial (**B**), and sagittal reformation (**C**) to quantify the included Hounsfield unit spectrum and to generate a 3D model to illustrate the extent of the odontogenic keratocyst (**D**). R = right; T = top; L = left; colored B = bottom; colored A = anterior; P = posterior.

DICOM data from the baseline CT scans of the participants included in the study were acquired and processed using Mimics image processing software (Mimics Innovation Suite 21.0, Materialise, Leuven, Belgium). For the measurement of lesions, CT images were color coded to distinguish the affected areas from the unaffected soft tissue and bony parts of the scanned jaw region. The differentiation was based on thresholding of HU using the above-mentioned software. Subsequently, a virtual tissue dissection was manually performed to correct the erroneously included structures (Figure 2A–C). After this assessment of the lesion, the HU spectrum (mean, median, standard deviation, minimum, and maximum) was calculated in each case using a function implemented in the Mimics software. The measurement was performed analogously for the available CBCT scans with calculation of the GSV spectrum, according to the previous work and results of Reeves et al., Abe et al. and Razi et al. [27,28,29,30]. This procedure was performed by two independent investigators (J.T. and N.L.) and the calculated HU as well as GSV spectra were only compared within the same imaging modality, i.e., CT or CBCT.

### 2.3. Statistical Analysis

The statistical analysis was performed using IBM SPSS Statistics 26.0 (IBM Corp., Armonk, NY, USA) and GraphPad Prism 9.0 (GraphPad Software, La Jolla, CA, USA). The median (MED), mean (MV), standard deviation (SD) as well as minimum and maximum of HU and GSV measurements were calculated. An unpaired *t*-test was used to analyze the differences between the CT- and CBCT-based approaches. Correlations were analyzed by calculating the Pearson correlation coefficients between OKC and AM within CT and CBCT, respectively. *p* values < 0.05 were considered significant.

## 3. Results

### 3.1. Characterization of the Participant Cohorts

During the observation period of 16 years, 51 participants met the defined inclusion criteria. A total of 20 participants (39.22%) were female and 31 participants (60.78%) were male. Of these, OKC occurred in 17 females (42.50%) as well as 23 males (57.50%) and AM in 2 females (18.18%) as well as 9 males (81.82%). The overall mean age was 42.06 years (range: 9 to 89 years), OKC participants had a mean age of 38.74 years (range: 9 to 89 years) and AM participants were slightly older with a mean age of 55 years (range: 42 to 79 years). Within the cohort of 40 OKCs, 19 participants were examined by CT (47.50%) and 21 by CBCT (52.50%). Within the cohort of 11 AMs, 7 participants received CT scans (63.64%) and 4 participants received CBCT scans (36.36%). 2 of the lesions were located in the mandibular front (3.92%), 20 in the mandibular angle (39.22%), 17 in the mandibular corpus (33.33%), 2 in the ramus (3.92%), and 10 in the maxilla and maxillary sinus (19.61%). The distribution of the location of lesions was similar in the OKC and AM cohorts with the mandibular angle being the most frequent location, followed by the mandibular corpus and the maxilla and maxillary sinus. The mean value of all measured lesion volumes was 7179 mm^3^ (range: 433 to 48,601 mm^3^) and the mean pixel number was 160,831 pixels (range: 2142 to 895,726 pixels). The clinical characteristics of the participant cohort are presented in Table 1.

### 3.2. Hounsfield Unit Spectra Discriminates OKCs from AMs

**Figure 3:**
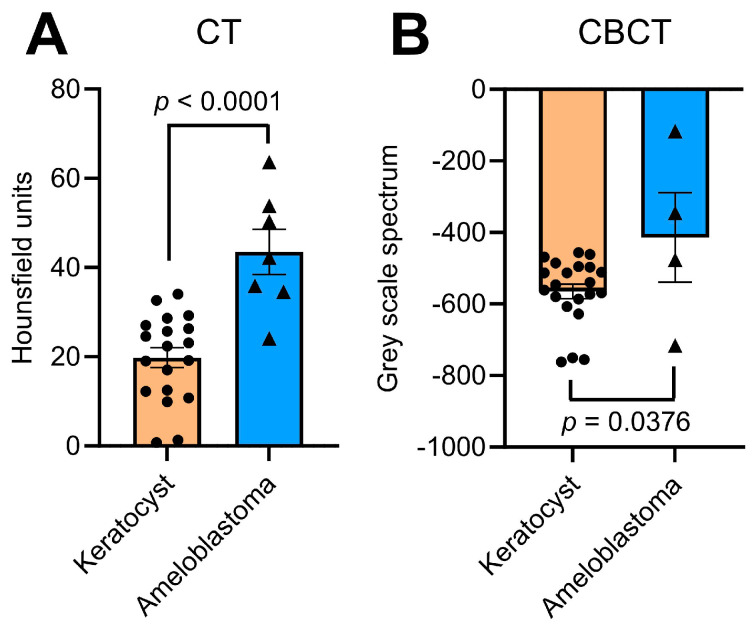
Differentiation of Hounsfield unit and gray scale spectra quantified for odontogenic keratocysts and ameloblastomas within imaging by CT (**A**) and by CBCT (**B**). Bars represent means ± SEM.

The measurements of HU and GSV spectra, respectively, performed independently by the 2 investigators for all lesions were similar and had a precise inter-rater reliability. The spectrum calculated on the basis of HU for OKCs in the CT scans averaged at 19.79 HU with a standard deviation of 9.73 HU (minimum 0.77 HU and maximum 34.04 HU). In contrast, the mean value for AMs was 43.52 HU with a standard deviation of 13.39 HU (minimum 24.10 HU and maximum 63.65 HU). Here, the unpaired *t*-test showed a highly statistically significant difference between the respective entities (*p* < 0.0001, Figure 3A, Table 2). Within the CBCT scans, the mean for OKCs was −564.76 GSV with a standard deviation of 93.08 GSV (minimum −762.39 GSV and maximum −455.96 GSV) and the mean for AMs was −413.76 GSV with a standard deviation of 250.69 GSV (minimum −716.63 GSV and maximum −116.71 GSV). Also, the unpaired *t*-test showed a statistically significant difference (*p* = 0.0376, Figure 3B, Table 2). These findings demonstrate that the HU and GSV spectra are significantly larger in AMs compared to OKCs indicating that the HU and GSV spectra can be used to discriminate between these two entities.

### 3.3. HU-Based Discrimination of OKCs and AMs Is Independent from Participant Characteristics

**Figure 4:**
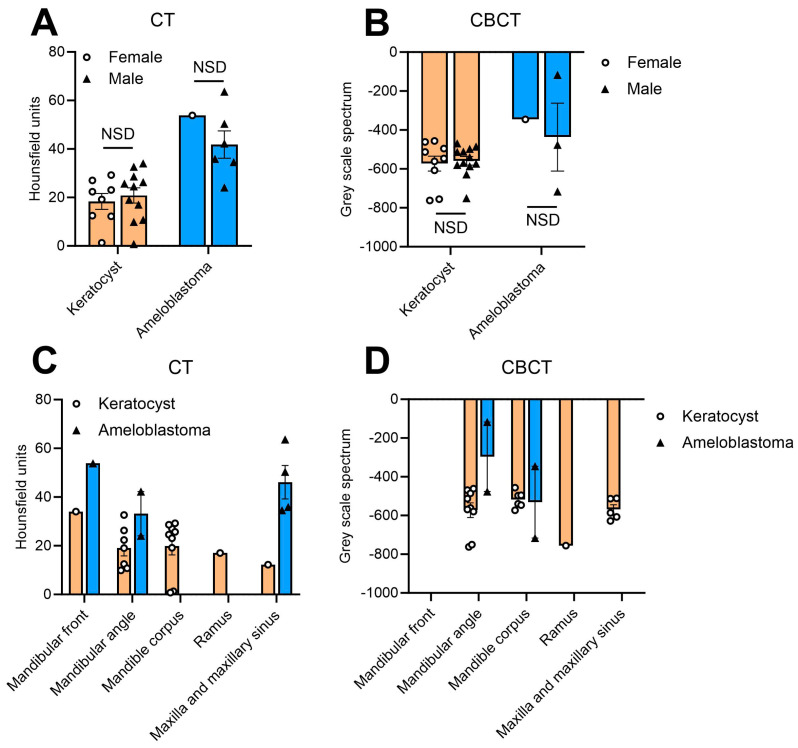
Hounsfield unit (HU) and gray scale (GSV) spectra quantified for odontogenic keratocysts and ameloblastomas. (**A**) HU within CT images of female and male participants. (**B**) GSV within CBCT images of female and male participants. (**C**) HU of odontogenic keratocysts and ameloblastoma within CT images depending on anatomical location. (**D**) GSV of odontogenic keratocysts and ameloblastoma within CBCT images depending on anatomical location. Bars represent mean ± SEM. NSD = not statistically significant.

The next step was to identify parameters which might impact the HU- and GSV-based discrimination between OKCs and AMs. When creating subcohorts based on participants’ gender, no significant differences were found for HU and GSV spectra neither within CT scans (Figure 4A) nor within CBCT scans (Figure 4B). Also, the above-described finding that AMs are characterized by increased HU and GSV spectra was observed independently of the participants’ gender (Figure 4A,B). The age of the participants did not impact the HU and GSV-based measurements since there were no significant correlations of the participants’ age with the HU and GSV (Table 3). Analogous, no differences were found for the HU and GSV spectra when comparing the HU and GSV of the lesions at different locations. HU in CT and GSV in CBCT scans were consistent at all anatomical sites and no significant differences were observed (Figure 4C,D). Lastly, the HU and GSV spectra were correlated with the measured volume of the lesions as well as with the measured number of pixels to evaluate whether the HU or GSV spectra are dependent on the size of the lesion and, therefore, might change during disease progression. No significant differences were observed (Table 3), indicating that the differences of HU and GSV between OKC and AM are not based on the size of the lesion, but reflect an individual configuration of the lesion.

## 4. Discussion

Early diagnosis of OKCs as well as AMs is critical for therapeutic decision making to ultimately improve treatment outcomes. An early and correct diagnosis of the specific lesion is crucial for the subsequent, in parts different, treatment strategy [1]. Identification at an early stage can spare the patient a great deal of suffering and, for example, make partial resection of the affected portion of the jaw unnecessary as part of the surgical treatment. Radiographic imaging plays a crucial diagnostic role for OKCs and AMs since most lesions are diagnosed as incidental findings [1]. However, the radiographic appearance of OKCs and AMs is similar, making differential diagnosis difficult. Conventional panoramic radiographs provide a two-dimensional view of the jaw, but more detailed imaging is required to accurately assess the size, location, and extent of the lesion. CT has been shown in previous studies to be beneficial and superior to panoramic radiographs in the detection of lesions of the jawbone [11,23,31,32,33]. Some preliminary work described CT values of such lesions [11,32,33,34], but only one study addressed the exact CT density values [26]. As far as the literature search revealed, an evaluation of the density values of OKCs and AMs within a CBCT has not yet been performed. Yet a CBCT in particular, as an imaging modality, represents an important link between a panoramic radiograph as well as CT, since it is often used by dentists in the diagnosis of various pathologies and is also lower in radiation exposure as well as less expensive for the patient [35,36].

In this study, a HU and GSV spectrum was determined for OKCs and AMs in CT and CBCT scans, respectively. Partially consistent with the previous work of Rebello et al. where only axial slice images were used without segmentation of the whole CT data, this work was able to differentiate both entities in the scans based on density values with a statistically significant difference in CT as well as CBCT [26]. Rebello et al. as well as Uehara et al. described values ranging from 28.4 HU to 37.9 HU in CT scans for OKCs and an average value of 35.9 HU for AMs, with which the results of this work barely agree, showing a mean range of 19.79 HU for OKCs as well as a mean value of 43.52 HU for AMs in the CT scan, thus showing a lower density for OKCs [26,37]. Furthermore, CBCT scans could reveal mean values of −564.76 GSV for OKCs and −413.76 GSV for AMs and thus a higher density for AMs compared to OKCs.

Considering parameters that might affect HU- and GSV-based discrimination between OKCs and AMs, gender of participants showed no significant differences for HU and GSV spectra within CTs and CBCTs. Similarly, participant age showed no significant correlation between HU- and GSV-based measurements. Also, no differences were found in the localization of individual lesions, as HU and GSV were consistentin CT as well as CBCT. The fact that the majority of both lesions were located in the posterior mandibular region is in accordance with previous studies [7,38,39]. When HU and GSV spectra were correlated with lesion volume and measured pixel number, no significant difference was found either. Since no studies could be found so far that had examined these parameters with the differences in HU and GSV spectra of both entities, this suggests that the density values of individual lesions are independent of sex, age, location, as well as bony extent, indicating an individual configuration of the lesion.

Since OKCs are cystic and have fluid-filled cavities, they can be expected to be less dense than AMs [11,31]. The cystic contents of OKCs contain low levels of soluble proteins and inflammatory exudates have been described with HU values of 18 HU [25,26]. Although values above 100 HU and described as areas of increased attenuation (IAA) have been reported in preliminary work, some factors such as the stage of the lesion or the presence of subepithelial inflammation may influence the occurrence of IAAs [26,33,40]. In contrast, in AMs, a previous work described areas with HU values similar to that of muscle tissue [34]. Furthermore, the strong vascularization of AMs was studied on CT and blood in turn showed HU values of 55.5 HU [17,25,26]. In general, AMs are composed of an epithelium and dense connective tissue, resulting in a higher average density [23]. The attenuation coefficients, which can be taken from CT scans, are able to describe different tissues numerically [26,41]. Multiple studies gave the recommendation to differentiate lesions such as adrenal adenomas from non-adenomas or adenocarcinomas of the lung from bronchioalveolar carcinomas based on their distinct density values [24,26,42].

This study had some limitations since not every OKC or AM participant necessarily received a CBCT scan or even a CT if the lesion size was manageable for subsequent surgical treatment. Furthermore, no lesion underwent both CT and CBCT, although this would have ensured direct comparability of the same lesion. In contrast to the clearly delineated HU area on CT, the spectrum of both entities on CBCT was shown to be partially overlapping and reflected a small significant difference. This may be due to the fact that older CBCTs were of lower quality and thus slightly distorted the results after segmentation. In addition, the sample size of the lesions investigated and the uneven distribution of OKCs to AMs is the major limitation of this work. CTs or CBCTs are still not the standard preoperative imaging technique for such lesions in our department, explaining the small cohort size included in this study. Standardized diagnostic use of both modalities should concretize this in future work by a larger collective. In addition, other imaging modalities such as MRI, PET/CT as well as PET/MRI should not be ignored, as they also represent an additional information gain, can improve the differentiation of both entities, and partly do not require radiation exposure [43,44,45,46,47]. However, from the perspective of a dental practice, the mentioned modalities are rather exotic imaging options that are currently rarely available in such outpatient settings.

In summary, the present study shows a, limited in its validity, good possibility to differentiate OKCs and AMs by their distinct density ranges in CT and much more importantly in CBCT. One of the main advantages of CBCT over conventional CT is the lower radiation exposure as well as low incurred costs. Also, like CT, it provides information about the relationship of the lesion to adjacent structures such as nerves and blood vessels, which is critical for minimizing the risk of complications during subsequent surgery. In addition, a CBCT can be performed in a dental office, which in turn is usually the first point of contact when an OKC or AM occurs. Since such lesions have so far been studied in conjunction with machine learning and neural networks using only panoramic radiographs and CTs [12,21], the use of such algorithms in CBCT scans offers an interesting approach for future studies.

## 5. Conclusions

The findings of this study demonstrate that CT-based differentiation between OKCs and AMs is feasible as a non-invasive diagnostic option. Similarly, differentiation using CBCT scans demonstrates another non-invasive, radiation-reduced and practicable possibility for the diagnosis of both entities.

## Figures and Tables

**Table 1:** Clinicopathological characteristics of the participant cohort.

Category	OKC (*n* = 40)	AM (*n* = 11)	Total (*n* = 51)
Gender:			
Female	17 (42.50%)	2 (18.18%)	20 (39.22%)
Male	23 (57.50%)	9 (81.82%)	31 (60.78%)
Age (MV in years)	38.74 (9 to 89)	55 (42 to 79)	42.06 (9 to 89)
Measurement Modality:			
CT	19 (47.50%)	7 (63.64%)	26 (50.98%)
CBCT	21 (52.50%)	4 (36.36%)	25 (49.02%)
Location:			
Mandibular front	1 (2.50%)	1 (9.10%)	2 (3.92%)
Mandibular angle	16 (40.00%)	4 (36.36%)	20 (39.22%)
Mandibular corpus	15 (37.50%)	2 (18.18%)	17 (33.33%)
Ramus	2 (5.00%)	0	2 (3.92%)
Maxilla and maxillary sinus	6 (15.00%)	4 (36.36%)	10 (19.61%)
Measured volume (MV in mm^3^)	4884 (433 to 19,678)	15,527 (2870 to 48,601)	7179 (433 to 48,601)
Measured number of pixels	140,733 (2142 to 895,726)	233,914 (19,415 to 850,491)	160,831 (2142 to 895,726)

OKC = odontogenic keratocyst; AM = ameloblastoma; MV = mean value; CT = Computed Tomography; CBCT = Cone Beam Computed Tomography. The range of age, measured volume and measured number of pixels is given in brackets.

**Table 2:** Analysis and differentiation of odontogenic keratocysts and ameloblasts using Hounsfield units and gray scale values within CTs and CBCTs.

	Measuring Modality			
	(*n* = 26)	CT	(*n* = 25)	CBCT
Entity	OKC	AM	OKC	AM
Mean	19.79	43.52	−564.76	−413.76
Median	22.38	42.26	−546.13	−410.84
SD	9.73	13.39	93.08	250.69
Minimum	0.77	24.10	−762.39	−716.63
Maximum	34.04	63.65	−455.96	−116.71
*p*-value	<0.0001		0.0376	

CT = Computed Tomography; CBCT = Cone Beam Computed Tomography; OKC = odontogenic keratocyst; AM = ameloblastoma; SD = standard deviation.

**Table 3:** Correlation of Hounsfield units and gray scale values with indicated parameters within CTs and CBCTs. Values represent Pearson correlation coefficients.

	Measuring Modality			
	CT		CBCT	
Category	OKC	AM	OKC	AM
Age	−0.018 *p* = 0.9433	0.661 *p* = 0.1063	−0.124 *p* = 0.6020	−0.909 *p* = 0.2737
Measured volume (mm^3^)	0.093 *p* = 0.7045	−0.410 *p* = 0.3613	−0.102 *p* = 0.662	−0.555 *p* = 0.4455
Measured number of pixels	0.264 *p* = 0.2747	−0.369 *p* = 0.4159	−0.057 *p* = 0.805	0.736 *p* = 0.265

CT = Computed Tomography; CBCT = Cone Beam Computed Tomography; OKC = odontogenic keratocyst; AM = ameloblastoma.

## Data Availability

Data can be obtained by scientists that conducted the work independently from the industry on request. Data are not stored on publicly available servers.
